# Supplementary material for: Quantitative modeling identifies critical cell mechanics driving bile duct lumen formation
Source: PLoS Comput Biol. 2022 Feb 18;18(2):e1009653. doi: 10.1371/journal.pcbi.1009653 (PMC8856558; doi:10.1371/journal.pcbi.1009653)
Supplement: S1 Text — (PDF) [file pcbi.1009653.s001.pdf]

Supplementary information 1: Model description

Paul Van Liedekerke, Lila Gannoun, Axelle Loriot, Frédéric P. Lemaigre, Dirk Drasdo

Basic DCM forces

In this section the force contributions acting on a cell within the deformable cell model (DCM) are summarized.

Elastic forces cortex

The cell surface in the DCM is triangulated with visco-elastic elements along the edges linking neighbouring nodes. The forces between the nodes mimic the cortical tensions. The membrane envelope is assumed to not significantly contribute to these forces as the presence of caveolae can significantly reduce its mechanical resistance [1]. We assume that cell deformation dynamics can be reasonably approximated by Kelvin-Voigt elements. The internal force F_{int} originating from a Kelvin-Voigt viscoelastic element between node nodes i and j reads:

$$\begin{aligned}\mathbf{F}_{int,ij} &= \mathbf{F}_{e,ij} - \gamma \mathbf{v}_{ij} \\ &= -k_s(l_{ij} - l_{ij}^0)\mathbf{e}_{ij} - \gamma \mathbf{v}_{ij},\end{aligned} \tag{1}$$

where γ denotes the friction coefficient, $l_{ij}^0 = ||\mathbf{r}_{ij}^0|| = ||\mathbf{r}_j^0 - \mathbf{r}_i^0||$ and $l_{ij} = ||\mathbf{r}_{ij}||$ are the initial (cell at rest) and actual lengths between the nodes, and $\mathbf{v}_{ij} = \mathbf{v}_j - \mathbf{v}_i$ is the relative velocity of nodes i, j. The force balance equation with external forces $\mathbf{F}_{ext}$ demands that $\mathbf{F}_{ext} + \mathbf{F}_{int} = \mathbf{0}$, hence:

$$\mathbf{F}_{ext,ij} - k_s(l_{ij} - l_{ij}^0)\mathbf{e}_{ij} - \gamma \mathbf{v}_{ij} = \mathbf{0}. \tag{2}$$

The linear spring constant for a sixfold symmetric triangulated lattice can be related approximately to the cortex Young modulus E_{cor} with thickness h_{cor} by [2]

$$k_s \approx \frac{2}{\sqrt{3}} E_{cor} h_{cor}. \tag{3}$$

Besides tension, the cortex resists to bending. The bending resistance in the cortex is incorporated by the angular resistance of the hinges determined by two adjacent triangles $T_1 = \{ijk\}$ and $T_2 = \{ijl\}$. This can be accounted for by the bending moment M:

$$M = k_b sin(\theta - \theta_0) \tag{4}$$

where k_b is the bending constant, and θ is is the angle between the normal vectors to the triangles $\mathbf{n}_\alpha, \mathbf{n}_\beta$ by their scalar product $(\mathbf{n}_\alpha \mathbf{n}_\beta) = cos(\theta)$. θ_0 is the angle of spontaneous curvature. A spontaneous curvature denotes the curvature for which the bending energy is zero.

The moment M can be transformed to an equivalent force system $\mathbf{F}_{m,z}$ $(z \in \{ijl\})$ for the triangles T_1 and T_2 where here for T_1 we can compute $\mathbf{F}_{m,i} = M/l_1\mathbf{n}_1$ using l_1 as the distance between the hinge of the triangle pair and the point i, and similar expression for $\mathbf{F}_{m,l}$. The forces working on nodes j, k must at least fulfill

$\mathbf{F}_{m,i} + \mathbf{F}_{m,j} + \mathbf{F}_{m,k} + \mathbf{F}_{m,l} = 0$ to conserve total momentum, see e.g. [3,4]. The bending stiffness of the cortex is approximated by

$$k_b \approx \frac{E_{cor} h_{cor}^3}{12(1-\nu^2)}, \tag{5}$$

where $\nu \approx 0.5$ is the Poisson's ratio of the cortex. Note that the elastic modulus of the cortex, E_{cor} enters both, the bending force and the tension force.

Volume forces

The volume change of the cell depends on the applied pressure and the cell bulk modulus K_V. The total compressibility of the cell depends on volume fraction of water in the cytosol, the cytoskeleton (CSK) volume fraction and structure, and the compressibility of the individual organelles. In addition, it may be influenced by the permeability of the plasma membrane for water, the presence of caveolae, and active responses inside and of the cell. We calculate the internal pressure in a cell by the logarithmic strain for volume change:

$$p = -K_V log(\frac{V}{V_0}), \tag{6}$$

whereby V is the actual volume and V_0 is the reference volume i.e., the volume of the cell not subject to compression forces. For small deviations of V from V_0, $p \approx K_V(V - V_0)/V_0$. Within our model the volume V of the cell is computed summing up the volumes of the individual tetrahedra that build up the cell. The nodal force is obtained by multiplying the pressure with the nodal Voronoi area S_i (see [5]), i.e. $\mathbf{F}_{vol,i} = pS_i\mathbf{R}_i$ where the local curvature vector $\mathbf{R}_i$ is computed for that node using a discrete Laplace-Beltrami operator [7].

Contact model and adhesive forces

Whereas in a CBM, cells interact through central forces described by (modified) Hertz or JKR theory for adhesive spheres, in DCM the interaction forces need to be defined for each node individually, thereby requiring a representation of local surface heterogeneities. The approach followed in this work adopts a discretised Maugis-Dugdale theory. The Maugis-Dugdale theory for adhering bodies is a generalization of the JKR theory for spheres [6]. This theory captures the full range between the Derjaguin-Muller-Toporov (DMT) zone of long reaching adhesive forces of a hard homogeneous isotropic elastic sphere and small adhesive deformations in the Johnson-Kendall-Roberts (JKR)-limit of a soft homogeneous isotropic elastic sphere of short interaction ranges. Here, we associate to each triangle of the cell surface a circumscribing sphere reflecting the local curvature. Two triangles belonging to different cells can locally interact by collision of their associated spheres. To compute the magnitude of these interactions with respect to the nodes of the triangles, we use the general Maugis-Dugdale stress, which is integrated over the common contact area between the triangles, resulting in the net forces on the nodes of the two interacting triangles (see [3,7] for more details). The adhesion force is fully determined by the specific adhesion energy W and the typical effective adhesive range h_0 that reflects the attractive cutoff distance between the bodies. We set $h_0 = 2 \cdot 10^{-8}m$ in all the simulations [8].

In our model, Maugis-Dugdale theory is applied to every set of triangles which constitute the cell surface. A varying number of cadherin bonds is mimicked by varying the adhesion energy along the cell surface triangles.

Migration forces

In models in which cell movement and deformation is mimicked by force-balance equations following Newton's law of motion as this is the case for the DCM and CBM, migration is usually modelled by an active migration force $\mathbf{F}^{mig}$ representing the random micro-motility of a cell. For the sake of simplicity the cell shape, filopodia etc. movements during migration are not detailed in the model but instead the different mechanisms in cell migration is lumped into one net force which is uniformly distributed to the nodes the cell if not mentioned otherwise. For specific applications, the forces might be non-uniformly distributed. In absence of influences that impose a certain direction or persistence, it is commonly assumed that the migration force is stochastic, formally resulting in $\mathbf{F}^{mig} = \mathbf{F}^{ran}$, with $\langle \mathbf{F}^{ran} \rangle = \mathbf{0}$, and $\langle \mathbf{F}^{ran}(t) \otimes \mathbf{F}^{ran}(t') \rangle = \mathbf{M}\delta(t - t')$, where $\mathbf{M}$ is an amplitude 3×3 matrix and relates to the diffusion tensor $\mathbf{D}$ of the cell. As cell migration is active, depending on the local matrix density and orientation of matrix fibers, the autocorrelation amplitude matrix $\mathbf{M}$ cannot a priori be assumed to follow a fluctuation-dissipation (FD) theorem. However, "measuring" the position of a cell in the simulations the position autocorrelation function might be experimentally used to determine the diffusion tensor using $\langle ((\mathbf{r}(t + \tau) - \mathbf{r}(t)) \otimes (\mathbf{r}(t + \tau) - \mathbf{r}(t)) \rangle = 6\mathbf{D}\tau$, and $\mathbf{M}$ be calibrated such that the numerical solution of the equation of motion for the cell position reproduces the experimental result for the position-autocorrelation function. For example, in a homogeneous environment $\mathbf{M}$ can be casted into a form formally equivalent to the FD-theorem, leading to an k_BT-equivalent for cellular systems, that is controlled by the cell itself [9, 10].

Note, we assume here a momentum transfer to the ECM by applying the ECM friction and active micro-motility term, but we do not model the ECM explicitly.

Cell cycle and cell division

During cytokinesis, the continuous shrinking of the contractile ring, together with the separation of the mitotic spindle, gradually divides the mother cell into two daughter cells. After mitosis the cell has split up in two adhering cells. Such a process of cell division in 2D deformable cells has been previously described (e.g. [11]) but it can be costly and tedious to perform in 3D.

As we are merely interested in long term effects (i.e. hundreds of cell divisions), and as the cytokinesis is a short process compared to the duration of the entire cell cycle, we avoid these particular tedious intermediate steps in our model, and directly create two new adhering cells that are within the shape of the mother cells just before its division using the basic algorithm established in ref. [12].

In our algorithm, the two created daughter cells are initially round and separated from each other by the division plane. They grow artificially fast [1] until they reach the boundary of the mother envelope. The division plane is removed from the system after the daughter cells have found a mechanical equilibrium in this envelope. We have here slightly extended our algorithm to mimic the very short rounding-up period just before division (see Fig 2G in main text). This is achieved by setting the equilibrium edge lengths (see below) of the triangulation temporarily to a smaller value. This can cause a slight increased pressure in the cell during division.

In the simulations, cells grow by increasing their volume and surface and they can divide when their actual volume reaches a target volume. This target volume is predefined and is for a newborn cell by default set to twice the volume of that newborn cell. The orientation of cell division can be chosen randomly (as would be the case for non polarized hepatocytes) or with a preferred direction. The latter is the case when a

[1] We call this a "sub-simulation" period, for which the time compared to the cell cycle is negligible.

polarized cell divides. In this case the division direction is the polarization vector. As observed experimentally, we also implement a temporary and short rounding up period just before cells division. This has a consequence that the cell volume slightly drops and hence the pressure inside the cell increases, causing a rounding of the surface. The shortening of the vertex edges is chosen such that this pressure increase is not more than 100 to 200 Pa, achieved by modifying the lengths from l_0 to $0.95 * l_0$. The rounding up period in our model does not take more than 5% of the total cell cycle time.

The cells in our system can either be proliferating or quiescent. Immediately after cell division, all cells are assumed to proliferate. A change to quiescence is here assumed to be induced by chemical signals of other cells. To ensure in the simulations that only the fraction of the cells is growing conform with the experimental observations, an algorithm is invoked that sweeps every time step over a certain cell type and ensures that the prescribed fraction of proliferating cells is maintained. The picking of cells that need to go to quiescence is done randomly. This algorithm was chosen as the mechanistic control of how many cells divide was not subject of the present simulation study.

DCM extension: Polarity vector

A normalized polarity vector $\vec{P}$ (PCP) is assigned to each of the cells. This vector defines for every cell two opposite conical polar regions (see Fig 2A in main text). The triangular regions on the cell surface that are marked as polar are defined by the scalar product condition:

$$\|\vec{P} \cdot \vec{N}_i\| < a, \tag{7}$$

where N_i is a normalized vector with origin the center of mass of the cell, and pointing to the middle of triangle i. The area of the polar region a can be chosen according to $0 < a < 1$. We chose in the simulations of this work $a = 0.7$. This corresponds to a bi-conical area with angle α of approximately 60 degrees. The polar regions (triangles) may have different physical properties than the rest of the cell surface, such as a different specific adhesion energy. The PCP vector is assumed to be perpendicular to the apico-basal vector, which is enforced in the model with every timestep. (We note here that in our planar simulations neither the PCP or ABP have a Z component.)

DCM extensions : apical vector, apical constriction and tight junctions

Every cell also has an apical vector $\vec{A}$ (ABP), which defines the apical and basal side of the cell. Similar to the polar regions, the apical and basal triangular regions on the cell surface are defined by the conditions:

$$\vec{A} \cdot \vec{N}_i > b \text{ (apical region)} \tag{8}$$

and

$$\vec{A} \cdot \vec{N}_i < -b \text{ (basal region)} \tag{9}$$

Like for the polar vector, this corresponds to a conical area with angle β and the scalar value of $b \in [0, 1]$ determines the area of the regions, however the difference is that the apical side of the cell may have different physical properties than the basal side. In this work the cortical cytoskeleton on the apical side can contract, causing apical constriction.

In the model, the apical vector is assumed every time step to point towards the principal region where the cell surface has the most free area, in line with the

assumption made that the apical side forms the cell's interface with the lumen. Because for every triangle of a cell the free area and contact area is known after applying the contact model, the principal vector $\vec{A}$ can obtained by

$$\vec{A} = \sum_i \vec{N}_i / \| \sum_i \vec{N}_i \|. \tag{10}$$

Here the summation only counts triangles that have a very small contact area with a triangle from another cell. These conditions can for example be fulfilled when gaps between cells are created due to osmotic effects (e.g. onset of a small cavity). $\vec{A}$ is updated every timestep, except during cell division and a short relaxation phase right after cell division.

Adherent cells can also develop tight junctions (TJ) between them. In the model, we define for every cell that has an apical vector, a region with TJ triangles i that fulfill the condition :

$$\vec{A} \cdot \vec{N}_i > c_1 \text{ and } \vec{A} \cdot \vec{N}_i < c_2 \tag{11}$$

Here c_1 and c_2 are the limit values that define a conical ribbon along the cell where TJ can be present (see Fig 2D in main text). The parameter values for c_1 and c_2 are chosen such that the ribbon is about 1 or maximum at 2 triangles thick. As TJs denote areas on the cell surface with a reinforced connection, a larger specific adhesion energy is assigned to these areas than to the other surface areas. As such, when two triangles of different cells with a TJ come into contact, the force necessary to separate them will be much higher than for normal cell-cell contacts.

The apical constriction on the apical side is modeled as a shortening of the equilibrium element lengths between connected nodes that are located within the apical region (see below). This results in a movement of the two nodes towards each other. The shortening operation is executed only once. After a certain relaxation time a new force equilibrium is reached between the nodes. The modification of the equilibrium element lengths of the nodal springs during apical constriction are computed assuming two different zones:

$$l_0 := l_0 * d_2 \text{ for: } \vec{A} \cdot \vec{N}_i > c_2 \text{ and } \vec{A} \cdot \vec{N}_i < c_1, \tag{12}$$

which is the zone of the tight junctions, and

$$l_0 := l_0 * d_1 \text{ for: } \vec{A} \cdot \vec{N}_i > c_1. \tag{13}$$

in the apical domain.

To simulate the presence of an apical circumferential ring, the strongest constriction is applied in the zone of the TJ ribbon, whereas a lower constriction is applied in the apical domain ($0 < d_2 < d_1 < 1$). We have adopted the values $d_2 = 0.5$ and $d_1 = 0.7$. This warrants a strong contraction[2]. Moreover, the adhesive energy on the apical side is assumed to be lower than elsewhere on the cell because generally one observes that cadherin staining is less strong there (see Fig A1B).

DCM extensions : Osmotic effects and signalling

We assume that osmotic forces in the system result from differences of solutes e.g by molecules such as salts that are released from the cell into the extracellular space. These molecules further diffuse locally and cause a concentration gradient with more

[2]The volume forces then stretch the basal side to balance the contraction of the apical side. An elongation of the lateral springs maybe envisaged to ensure that the cellular pressure maintains its original value.

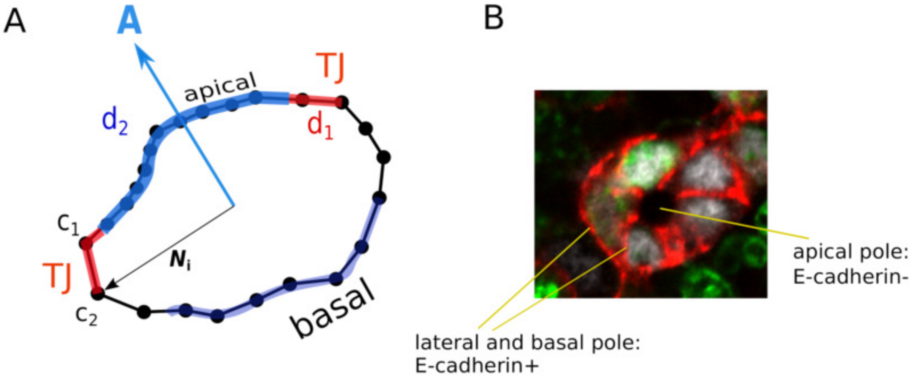

Figure A1. Cartoon indicating the parameters needed to determine the apical domain and apical circumferential ring.

distant locations. For example, if one cell starts to excrete ions, a salt concentration gradient is generated attracting water molecules towards the cell potentially causing a hydrostatic pressure increase strong enough to push away cells in the direct neighborhood of the central cell. As a consequence an extracellular space can arise, depending in shape and size on the dynamics and mechanics of the surrounding cells. One way to simulate the diffusion process of salts and flow of water could be to solve a system of partial differential equations (PDEs) for diffusive transport of salt and the advection of water. As the surrounding cells reorganise during the lumen formation process and hence the shape of the extracellular space changes, the boundary conditions for the PDEs change constantly. The constant change requires high resolution meshing and constant re-meshing of the complex cavity, which is numerically complicated and computationally time consuming. To alleviate this, inspired by smoothed particle hydrodynamics methods [13], we introduce the concept of "tracer particles (TP)", which are small "inert" particles. They can diffuse freely inside the cell or in the extracellular space without mutual interaction (see Fig 2F in main text). All the tracer particles (we used about 500 in numbers per cell) are initially inside the cell and start diffusing from there. The advantage of using particles to mimic the fluid is that their motion can be simulated by a Newton's equation of motion as for the cells, hence the coupling of fluid movement and cell deformation is straightforward.

The force on each particle originates principally from Brownian motion as and its magnitude is controlled by the TP diffusion coefficient. Additionally, a small force component is exerted to them in the apical direction to facilitate their movement towards the apical side which can be justified by directional vesicle transport towards lumen in polarized cells [14].

To ensure that the TP can leave the cell only through a certain region, we define a "transparent" region for each cell surface through which these particles can move into the extracellular space. The transparent region is equal to the apical side of the cells. In other parts of the cell, the particles cannot move across the cell surface. The particles can thus locally cross the cell boundary when moving from inside to outside, but are always repelled when trying to move back from the outside to the inside of the cell. These particles do not represent individual ions but rather each population of ions approximately obtained by integrating the spatial density of ions over local space regions. Nevertheless, to ensure diffusion constant is similar to that of the ions, the

particles are assigned with the diffusion coefficient of the ion species.

In the extracellular space a tracer particles marks a triangle if it is in close proximity of that triangle representing a free cell surface piece, not in full contact with another cell. As there are many TP present, all free surface areas of a cell will progressively become marked. To simulate the resulting hydrostatic pressure on that cell, each marked triangle receives an external force $\vec{F}_{osm} = PA_i\vec{n}_i$, where P is the assumed hydrostatic pressure that corresponds to the osmotic pressure generated by the difference in ion concentration (assumed constant here), and A_i is the surface of the marked triangle with local normal $\vec{n}_i$. Triangles belonging to two different cells that are in contact with each other have a negligible probability to be marked as the particles cannot access the space between them.

CBM forces

The CBM does not resolve cell shape explicitly. Forces are in the CBM are assumed to be exerted on the center of mass of the cell.

Adhesive and repulsive forces

In the CBM, cells are approximated by homogeneous, isotropic, elastic and adhesive spheres which split into two adherent cells during mitosis. Under conditions met in this work [16, 17], the total cell to cell interaction force can be approximated by the JKR force. The interaction force is computed by

$$Fcc, ij = \frac{4\hat{E}}{3\hat{R}}\left[a(\delta_{ij})\right]^3 - \sqrt{8\pi\sigma\hat{E}\left[a(\delta ij)\right]^3}. \tag{14}$$

The contact radius a in Eq. 14 if a function of the overlap $\delta_{ij} = ||\vec{r}_j - \vec{r}_i|| - R_i - R_j$ between the cell, allows to compute the cell-cell contact area, and can be obtained from:

$$\delta_{ij} = \frac{a^2}{\hat{R}} - \sqrt{\frac{2\pi\sigma}{\hat{E}}a}. \tag{15}$$

In the latter equations, $\hat{E}$ and $\hat{R}$ are defined as

$$\hat{E} = \left(\frac{1-\nu_i^2}{E_i} + \frac{1-\nu_j^2}{E_j}\right)^{-1} \text{ and } \hat{R} = \left(\frac{1}{R_i} + \frac{1}{R_j}\right)^{-1},$$

with E_i and E_j being the cell Young's moduli, ν_i and ν_j the Poisson numbers and R_i and R_j the radii of the cells i and j, respectively.

To enforce consistency with the by construction more accurate DCM that explicitly accounts for multi-body interactions, the Young modulus for every cell E_i in the JKR model was replaced by an "apparent" Young's modulus $\tilde{E}_i$ that increases as function of the local cell density [12]:

$$\tilde{E}_i = E_i + a_0\tilde{d}_i + a_1\tilde{d}_i^2 + a_3\tilde{d}_i^3 + a_4\tilde{d}_i^4$$

Here, $\tilde{d}_i =< 1 - ||\vec{r}_j - \vec{r}_i||/(R_{ref,i} + R_{ref,j}) >$ is the dimensionless average cell-cell distance. The parameters a_i can be determined in a calibration experiment, where one compresses a spheroid and measures the force between the cells [1, 12].

Because the CBM and the DCM are used in hybrid mode, they can make contact with each other. Using the Maugis-Dugdale formulation this can be solved in a natural way. The contact between a CBM and a triangle of the DCM is resolved using the triangle-triangle contact formulation for two DCMs, yet one of the triangles circumscribing sphere is here replaced by the sphere of the CBM cell itself. When the contact force is calculated, it is for the CBM directly passed to its center of mass.

CBM: Migration forces

The same principles for modeling the migration force in the DCM are applied to the CBM, whereby the migration force in the CBM is directly applied to the cell center.

CBM: Cell division

As in the DCM, if the cell passed a critical volume $V_{crit} = 2V_{0,i}$, the cell undergoes mitosis and two new cells with volume $V_{0,i}/2$ are created. A simplified version of the division algorithm consists of placing directly two smaller daughter cells in the space originally filled by the mother cell at the end of the interphase [15, 16]. When the two daughter cells are created, their reorganize in space driven by their cell-cell interaction force as well as the interaction forces of the two daughter cells with their other surrounding cells until mechanical equilibrium is reached. If the space filled by the mother cell is small, which is often the case for cells in the interior of a cell population, the local interaction forces occurring after replacing the mother cell by two spherical daughters, can adopt large (un-physiological) values leading to unrealistic large cell displacements. This can be circumvented by truncating the contact force to a maximal value, or temporarily reducing the contact stiffness between the daughter cells during division.

Cell-to-cell signalling

In our model, we assume that Notch-jagged signaling between cholangiocytes and hepatocytes is governed by several of conditions. First, we assume that their common contact area must be sufficiently high. Thus, a minimal number of triangles (or surface area) of both cells must be in mutual contact: $A_T > A_{min}$, where A_T is the total contact area between two cells, and the parameter A_{min} is set arbitrarily to 20% of the cell surface. We found that this parameter does not influence the results significantly provided that A_{min} is larger than zero. However, this condition alone is not enough for a hepatocyte to transform into a cholangiocytes, as then any hepatocyte in the tissue adjacent to a cholangiocytes would be able to transform at some point, causing a homogeneous spread of cholangiocytes, which is not observed experimentally. Hence, the second condition for the transformation is that the hepatocyte must delimit an existing (possibly small) lumen. Third, we also need to introduce a transition contact time T_{sig} which specifies the minimum time the signalling needs in order for a hepatocyte to change to a cholangiocyte. A value $T_{sig} = 0$ would mean here that the transformation is immediate, which is not observed as some hepatocytes may express weak SOX9+ signals but have a much larger size than a mature cholangiocyte. The signaling time is likely limited by the cell cycle time. When a hepatocyte gets the signal to transform, we assume that it will be fully differentiated upon the next cell division. We set here $T_{sig} = 2h$ as default value.

Initialization of model and boundary conditions of bile-duct system

The cells are initially positioned side by side on concentric circles with curvature determined by the radius of the portal vein. The most inner layer represents the portal vein endothelial for which we assume the positions are fixed during the simulations. The second layer are the mesenchyme, followed by the hepatoblasts, partially represented by the DCM and the outer layers fully represented by the CBM.

A background pressure P_b of the CBM hepatoblasts needs to be exerted at the outer border of the segment as a result of radial tissue growth and resistance from nearby

cells. This is achieved in the model by exerting an inward body force to the outermost border cells such that the inner hepatoblasts represented by the DCM have an average internal pressure of P_b. For the DCM, the pressure is calculated by Eq.6. For the CBM the pressure p_i on a cell i is derived from the virial stress and given by:

$$p_i = \frac{1}{3} tr(\sigma_i) \text{ , with } \sigma_i = \frac{1}{\mathcal{V}_i} \sum_j \left(\vec{F}_{ij} \otimes \vec{r}_{ij} \right) \tag{16}$$

being the stress tensor quantifying the stresses cell i experiences subject to contact forces $\vec{F}_{ij}$ with other cells j [9]. Here, $\vec{r}_{ij}$ is the vector pointing from the center of cell i to the cell j with $||\vec{r}_{ij}|| = d_{ij}/2$ and $\mathcal{V}_i$ is the *sampling* volume which can be taken as the cell volume.

To ensure the cells remain in a planar configuration, we apply a small penalty force on the center-of-mass of all the cells each time the center of mass moves away from the XY plane. A simulation then starts in which only the cells positions are updated and a mechanical equilibrium is reached. This configuration is then used for all further simulations.

Calculation of the bile duct lumen area

We obtain the lumen area by considering the triangles of the cells that are marked by tracer particles. The procedure to compute the lumen volume is conceptually similar to how the volume of a cell is computed given its triangulated structure (see ref. [12]). First, the geometric center of all these triangles is computed, resulting in a vector pointing to the geometric center of the bile duct. From this point, we compute the signed volume of each tetrahedron with as base a marked triangle and as top the geometric center. The sign here is determined by the normal vector of the triangle. This is done for all marked triangles, and subsequently these volumes are then summed up. This gives a reasonable estimation of the volume confined by the marked cells.

Computational scheme executed during simulation

The simulations have been performed in TiSim, a computational platform for tissue simulations based on agent-based models. This software will be released in the near future.

In every timestep, the new positions of the nodes of the DCM and the centers of the CBM are sought, given the forces that act on them. To compute these forces, one must execute a contact detection algorithm over the arrays of interacting objects to know which objects (triangles, spheres) can interact. This is a computationally expensive task which requires an effective method. We have here used a bounding box algorithm associated which each triangle and sphere. To compute the cell positions, the system of equations (1) and (2) in the main text are solved simultaneously. This is a linear system which can be solved efficiently by a conjugate gradient (CG) method. As the CG iterations are taking a large part of the computational time, especially as the system becomes larger, we have parallelized some loops in the CG algorithm using OpenMP. The optimal number of threads is here typically 2 to 3. After the velocities are calculated, we use an Euler scheme to obtain the positions. We have used in all simulations a stable timestep of 1s. The full bile duct system of cells after 24h of simulation time contains about 20000 nodes from the DCM, 500 nodes from the CBM, and 7500 tracer particles. The time so solve this system takes about 48h on a modern machine (Intel(R) Xeon(R) Gold 6136).

Based on the cell positions and forces, actions are undertaken in the algorithm. In Fig A2 , a model flow chart is given with the principal algorithms that are executed

every timestep. The algorithms that are cell -type dependent, are colored according to the cell type.

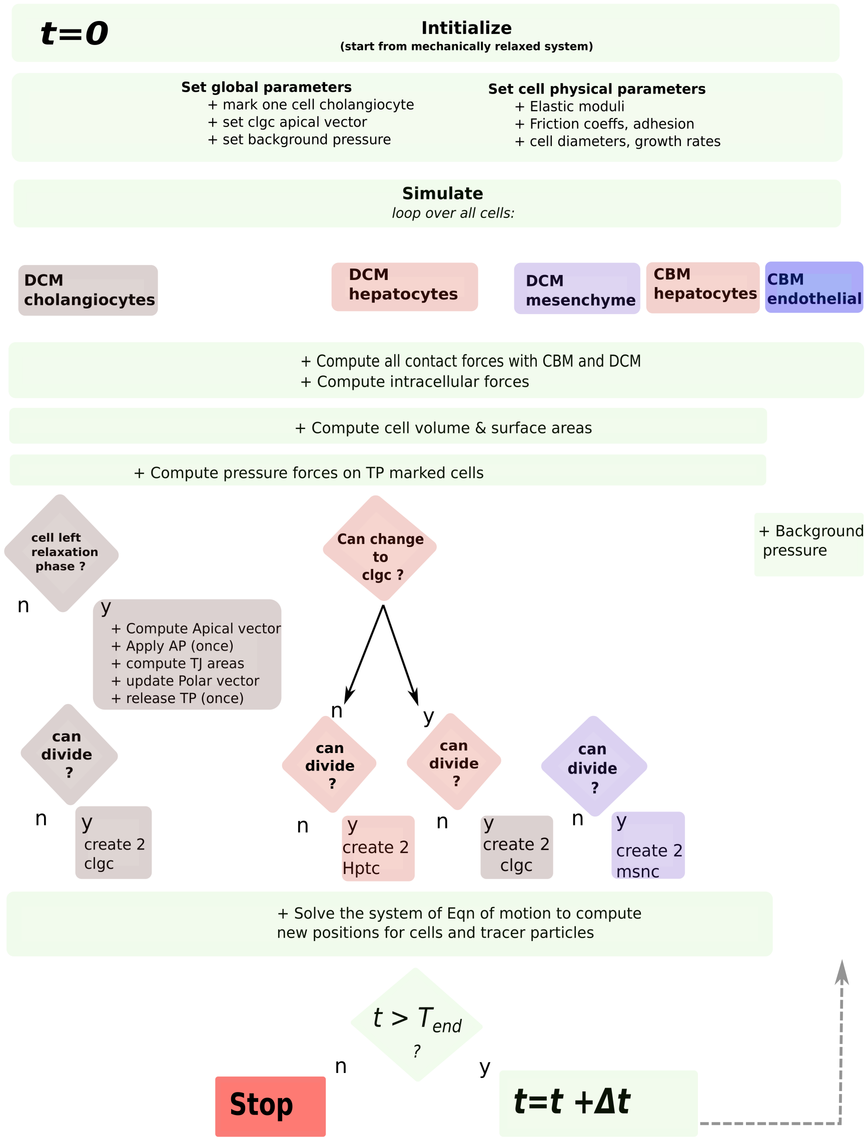

Figure A2. Computational scheme executed during simulation.

References

1. Van Liedekerke P, Neitsch J, Johann T, Alessandri K, Nassoy P, Drasdo D. Quantitative agent-based modeling reveals mechanical stress response of growing tumor spheroids is predictable over various growth conditions and cell lines. PLoS Computational Biology. 2019;doi:10.1371/journal.pcbi.1006273.

2. Boal D. Mechanics of the Cell. 2nd ed. Cambridge University Press; 2012.

3. Smeets B, Odenthal T, Keresztes J, Vanmaercke S, Van Liedekerke P, Tijskens E, Saeys W, Van Oosterwyck H, Ramon H. Modeling contact interactions between triangulated rounded bodies for the discrete element method Computer Methods in Applied Mechanics and Engineering; 2014, 227

4. Smeets B, Cuvelier M, Pešek J, Ramon H. The Effect of Cortical Elasticity and Active Tension on Cell Adhesion Mechanics. Biophys J. 2019. doi:10.1016/j.bpj.2019.01.015

5. P. Van Liedekerke, A. Buttenschoen, and D. Drasdo, "Off-lattice agent-based models for cell and tumor growth: Numerical methods, implementation, and applications," in *Numerical Methods and Advanced Simulation in Biomechanics and Biological Processes*, Miguel Cerrolaza Sandra Shefelbine Diego Garzón-Alvarado, 2017.

6. Maugis D Adhesion of spheres: the JKR-DMT transition using a Dugdale model. J Colloid Interface Sci 150(1):243–269

7. Odenthal T, Smeets B, Van Liedekerke P, Tijskens E, Van Oosterwyck H, Ramon H. Analysis of Initial Cell Spreading Using Mechanistic Contact Formulations for a Deformable Cell Model. PLoS Computational Biology. 2013;9(10):e1003267. doi:10.1371/journal.pcbi.1003267.

8. Leckband D., Israelachvili J. Intermolecular forces in biology. Q Rev Biophys 34(2):105–267 2001

9. Van Liedekerke P, Palm MM, Jagiella N, Drasdo D. Simulating tissue mechanics with agent-based models: concepts, perspectives and some novel results. Computational Particle Mechanics. 2015;2(4):401–444. doi:10.1007/s40571-015-0082-3.

10. Drasdo D, Hoehme S. Modeling the impact of granular embedding media, and pulling versus pushing cells on growing cell clones. New Journal of Physics. 2012;14(5):55025.

11. Jamali Y, Azimi M, Mofrad M RK, A sub-cellular viscoelastic model for cell population mechanics PLoS One. 2010; doi:10.1371/journal.pone.0012097

12. Van Liedekerke P, Neitsch J, Johann T, Warmt E, Gonzàlez-Valverde I, Hoehme S, et al. A quantitative high-resolution computational mechanics cell model for growing and regenerating tissues. Biomechanics and Modeling in Mechanobiology. 2020;doi:10.1007/s10237-019-01204-7.

13. Heck T, Vargas DA, Smeets B, Ramon H, Van Liedekerke P, Van Oosterwijck H. The role of actin protrusion dynamics in cell migration through a degradable viscoelastic extracellular matrix: Insights from a computational model. PLOS Computational Biology 16(1): e1007250. https://doi.org/10.1371/journal.pcbi.1007250

14. Benhamouche-Trouillet S, O'Loughlin E, Liu CH, Polacheck W, Fitamant J, McKee M, et al. Proliferation-independent role of NF2 (Merlin) in limiting biliary morphogenesis. Development (Cambridge). 2018;doi:10.1242/dev.162123.

15. Schaller G, Meyer-Hermann M. Multicellular tumor spheroid in an off-lattice Voronoi-Delaunay cell model. Phys Rev E. 2005;71(5):51910. doi:10.1103/PhysRevE.71.051910.

16. Galle J, Loeffler M, Drasdo D. Modeling the effect of deregulated proliferation and apoptosis on the growth dynamics of epithelial cell populations in vitro. Biophysical journal. 2005;88(1):62–75.

17. Drasdo D, Hoehme S, Block M. On the Role of Physics in the Growth and Pattern Formation of Multi-Cellular Systems: What can we Learn from Individual-Cell Based Models. Journal of Statistical Physics. 2007;128:287–345.

18. Hoehme S, Brulport M, Bauer A, Bedawy E, Schormann W, Hermes M, et al. Prediction and validation of cell alignment along microvessels as order principle to restore tissue architecture in liver regeneration. Proceedings of the National Academy of Sciences. 2010;107(23):10371–10376.
