# Supplementary material for: Quantitative modeling identifies critical cell mechanics driving bile duct lumen formation
Source: PLoS Comput Biol. 2022 Feb 18;18(2):e1009653. doi: 10.1371/journal.pcbi.1009653 (PMC8856558; doi:10.1371/journal.pcbi.1009653)
Supplement: Attachment — Submitted filename: response_reviewer_comments.pdf [file pcbi.1009653.s010.pdf]

Reviewer #1:
Page numbers used below are for the manuscript (shown at bottom of page as e.g., 12/30), not the page number in the PDF.

Overall, the work is well thought out and implemented, and addresses interesting issues in lumen formation in bile ducts in developing liver. The manuscript is well structured though clearly not written by a native English speaker. The language is often difficult to follow, or the word choice is poor.

Main Manuscript
Throughout the document the quote marks are incorrect. If this was typeset in LaTex then the proper quoting characters are on the left `` (two apostrophes) and on the right ‘’ (two single quotes). There are other quoting methods available in latex to give the proper “smart quotes” that point in the proper direction.

Thank you, this has been corrected in the text.

A brief discussion in the “Computational Models” section describing these models as being “2.5D” would be helpful. It is clear in the supplement (and parameter table, and suggested by the figures) that the model is 3D but consists of a single layer of cells constrained to not migrate from the XY plane, but that should be made clearer in the main manuscript.

This has been modified. See page 7.

Page 4
Near lines 57-60. Should cite http://www.ncbi.nlm.nih.gov/pmc/articles/pmc5221598/ as an example of a spatially defined model where a cavity forms, in this paper it is a cyst but the early process appears relevant to bile duct formation.

The reviewer is right, we have integrated this reference in the Introduction

Line 65: “aforementioned conditions” is unclear, multiple things have been described in the previous paragraph. Which specific conditions?

This has been modified the text. See page 4.

Line 83: In this work, we have put centrally three different mechanisms that are hypothetised to contribute to lumen formation. -> In this work, we have included three different mechanisms that are hypothesized to contribute to lumen formation.

This has been modified the text. See page 4.

Line 87-: In a second stage, guided by the 87 quantification of morphological features and expression of genes in developing bile ducts 88 specifically in embryonic mouse liver we constructed an in silico system representing a 89 part of the lobule containing the portal vein and surrounding tissue (Fig 1B, D). -> In a second stage, guided by the 87 quantification of morphological features and expression of genes in developing bile ducts 88 specifically in embryonic mouse liver, we constructed an in silico system representing a 89 portion of the lobule containing the portal vein and surrounding tissue (Fig 1B, D).

This has been modified the text. See page 4.

Page 6

Table 1: the second row of the table appears to list two different antibodies or perhaps the maker is Merck-Millipore?

The maker is indeed Merck-Millipore. The table 1 (p6) has now been corrected:

SOX9	Rabbit	Merck Millipore	AB5535	1/250

Page 7

Line 154-155: sentence is poorly written.

This has been modified the text. See page 7.

Lines 161-166: besides division on a random axis, or related to a polarization axis, what about division perpendicular to the long axis of the cell? A growing cell will often expand in the direction of least resistance giving an elongated cell that generally divides perpendicular to the long axis.

Fig. 3A shows that the longest possible axis of division would be from a basolateral to the contralateral apicolateral angle. Division perpendicular to such axis would generate a multilayered epithelium lining the duct. Division parallel to the apico-basal axis would also generate a multilayered epithelium. Instead, division perpendicular to the apico-basal axis would produce two daughter cells that maintain

the epithelium single-layered. See figure below.This is observed also in other developmental stages in which a cell layer is maintained, as during cleavage in early development of the sea urchin to form a hollow blastula (Wolpert and Gustafson, Experimental cell Research 25,374-382, 1961). Therefore, we assumed a division maintaining the one-cell-thick layer. In support of this assumption, variable axis of division as seen in bile ducts from patients affected with polycystic kidney disease, generates multilayered and irregular duct epithelium (Raynaud et al. Hepatology 53, 1959-1966, 2011). Therefore, we believe that our assumption which opposes division on a random axis or polarization axis is in line with the tissular architecture. We comment on this in the revised version. See page 9.

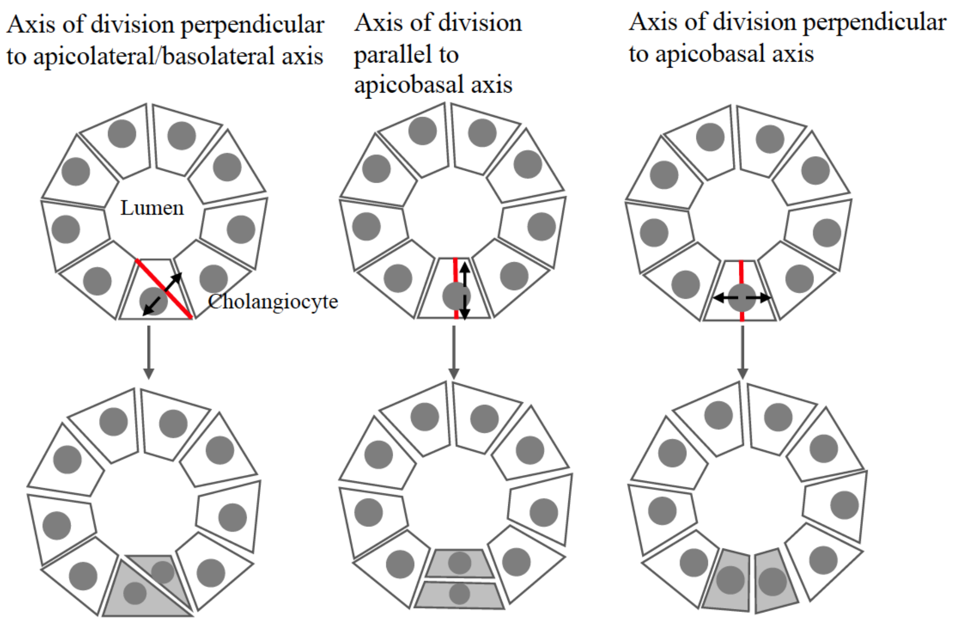

Lines 167-168: very poorly worded and unclear. “compartments” means “cells? “mostly be readily be estimated” should be “mostly be readily estimated”?

This has been modified the text. See page 9.

Lines 174-175: the polarity vector may not align with a reasonable cell division plane (see previous comment).

This comment overlaps with the reviewer’s previous comment on lines 161-166 (axis of cell divisions). Please see our reply above.

Page 8

Fig 2 caption: “mother envelope. upon confinement of the daughter cells,” is there missing text here? This can be written better.

This has been modified the text. See Fig. 2 page 8.

Page 9

Line 185 omit “bonds”

Line 193 shrinked -> shrank or reduced

This has been corrected in the text.

Line 200-202: sentence it unclear

This has been modified in the text. See page 9.

Line 209: extra space “Fig 2D) . The”

This has been corrected in the text.

Line 209: the “belt” region is part of the cell’s lateral surface.

This has been corrected in the text. See page 9.

Line 210: physical -> discrete

Line 211: “can be secreted by a cell Fig 2E .” à can be secreted by a cell (Fig 2E).

This has been corrected in the text. See page 9.

Line 214: pressures -> pressure

This has been corrected in the text. See page 9.

Line 215-217: unclear, perhaps “Conceptually similar, TP could also represent signaling molecules that are sensed by a nearby cells.”

This has been corrected in the text. See page 10.

Line 215-217: I don't think you mention at what stage of development flow starts in both the portal vein and in the bile ducts. Is it possible the TP is bile or a similar material?

At the onset of bile duct lumen formation (E15.5) the hepatocyte canaliculi are not yet connected to the developing bile ducts. Imaging of nascent canaliculi at E17.5 is illustrated by Belicova *et al.* (Belicova et al. J. Cell. Biol. 2021 Oct 4;220(10):e202103003.). Therefore TP's do not correspond to bile when bile duct lumen formation is initiated. When lumina have formed, bile may contribute to lumen expansion: work by Tanimizu *et al.* (Hepatology 2016; 64,175-188) shows that bile is detected in the intestine of E17.5 embryos, indicating that bile flows at this stage. We now mention this information and cite Tanimizu's paper in the revised version (line 224). In addition, we have evidence that a fluorescent bile acid (cholyl-lysyl-fluorescein) administered to pregnant females is transferred via the placenta to the embryonic hepatocytes and becomes detectable in the embryonic canaliculi and subsequently in bile ducts starting only around E16.5. To illustrate this, we here provide the reviewer with a figure, which we do not incorporate into the present paper, since we wish to keep it for a manuscript describing the molecular mechanisms driving formation of bile duct-hepatocyte canaliculi junctions.

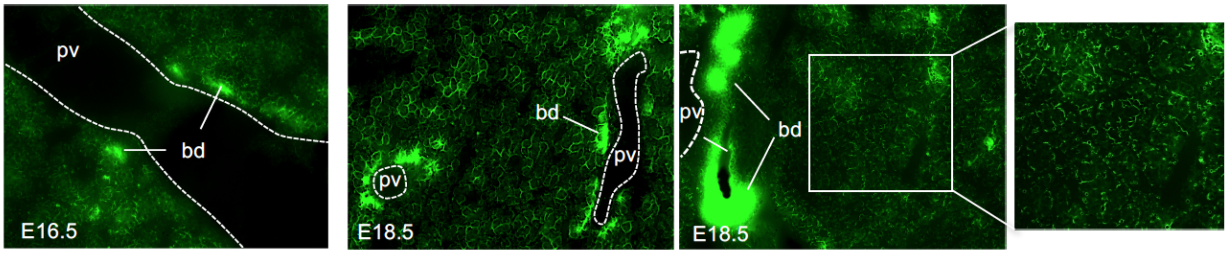

CLF detected in nascent canaliculi and bile ducts, 15 min after injection

CLF detected predominantly at basal pole of hepatocytes, also in canaliculi and bile ducts,10 min after injection

CLF detected in canaliculi and bile ducts,15 min after injection

Figure. Pregnant mice at E16.5 or E18.5 were injected with cholyl-lysyl-fluorescein (CLF), and embryos were collected 10 and 15 min after injection. Embryonic livers were dissected and imaged to detect the location of CLF. bd, bile duct; pv, portal vein.

Further, histologically stained sections through the embryonic liver, at any stage of development, show that the blood vessels contain red blood cells, indicating that the vessels are indeed perfused. This is well illustrated in Crawford et al. Toxicologic Pathology 2010; 38:872. *Histology atlas of the developing mouse hepatobiliary system with emphasis on embryonic days E9.5-E18.5*. We now mention this information further in the revised text, in reply to the reviewer's comment on page 10.

Page 10
Table 2: “personal communication” generally includes the person’s name, which may be one of the authors, or someone unrelated to the work in question. Perhaps replace with “estimated”?

Estimated is indeed a more appropriate term and we corrected the table. Desai et al. (Hepatology. 2016; 64: 261–275) determined a physiological liver matrix stiffness around 150 Pa, but this value is for adult liver. Our colleague Adrian Ranga, expert in tissue engineering, suggested a value or 50 Pa for embryonic liver; his name is mentioned in the acknowledgements.

This is clarified in page 20.

Line 240: Use of a period in “lhs.” and “rhs.”. Here there are periods but earlier without, see lines 149 and 151 where it is “rhs”.

This has been corrected in the text.

Page 11
Line 250: extra)

This has been corrected in the text.

Line 263: “Beta-catenin is here used as epithelial marker.” -> “Beta-catenin is here used as an epithelial marker.”

This has been corrected in the text.

Page 12
Line 294: difference may be responsible fot a lumen formation. -> difference may be responsible for lumen formation.

This has been corrected in the text, page 12.

Line 300-302: poorly written sentence. Perhaps “The heterogeneous morphology and circumferential expression of E-cadherin in hepatoblasts did not allow us to delineate the apical and basal sides or measure the length of the poles.”

This has been corrected in the text, page 12

Page 13
Line 306: “side of the cell” -> “side of the cholangiocyte cells”

This has been corrected in the text, page 13.

Line 307: as mentioned earlier, possible early secretion of bile micelles instead of salts? Fluorescein is an excellent marker of bile and brightly labels bile canaliculi in mature rodent livers. (https://pubmed.ncbi.nlm.nih.gov/25339682/) Have mice livers of this stage of development been examined for bile formation?

Line 321: Is it possible that flow has begun from bile canaliculi precursors?

This question overlaps with the reviewer’s question on lines 215-217. Please see our answer above which illustrates bile flow through the canaliculi and bile ducts using cholyl-lysyl-fluorescein.

Figure 4: bars are StDev? Range?

The bars are standard deviation. This is now mentioned in the revised figure legend.

Page 14
Line 348 Inconsistent use of a period in “Fig. 1A”

This has been corrected in the text, page 15.

Line 355: “Four cells in the upper layer are selected, able to proliferate.” -> “Four cells in the upper layer are selected as being able to proliferate.”

This has been corrected in the text, page 15.

Line 371: “chosen to be random corresponding to a uniform distribution” -> “chosen from a random uniform distribution”

This has been corrected in the text, page 16.

Page 16
Line 388 “with apical constriction, also Tight Junctions (TJ) are present.” -> “with apical constriction, Tight Junctions (TJ) are also present.”
Line 396 “forming” à “formed”

This has been corrected in the text, page 16.

Page 17

Lines 429-433: perhaps “Here the presence of tight junctions between the cells helps prevent bursting as they reinforce the cell-cell adhesion and provide a larger mechanical resistance to osmotic forces. Moreover, the tight junctions prevent ions from moving through the cell-cell boundary regions thereby hindering leakage out of the formed cavity.”

This has been adapted in the text, page 17.

Line 438: omit the first “different”.
Line 439: “hypothesised mechanisms in an idealized system with no external constraints to a” -> “hypothesised mechanisms in an idealized system, with no external constraints, to a”.
Line 440: “it is studied” -> “we examine”

This has been corrected in the text, page 17.

Line 443: “aligning” -> “lining”

This has been corrected in the text, page 17.

Line 444: “cues such” -> “cues, such”
Line 445: “signalling has” -> “signalling, has”
Line 457: omit “cut”
Line 459: “warranted”-> “obtained”
Line 461: omit “later forming the portal triads”
Line 462 “to” -> “of”

This has been corrected in the text, page 17.

Line 472: “aligning” à “lining” (this occurs in a couple places)

This has been corrected in the text, page 18.

Line 473-474: is there blood flow in the portal vein at E16.5 – E18.5?

As mentioned above, histological sections of embryonic livers (e.g. Crawford et al. *Histology atlas of the developing mouse hepatobiliary system with emphasis on*

embryonic days E9.5-E18.5.) illustrate the presence of red blood cells in the blood vessels. This is now mentioned in the revised text, page 18

Page 18
Line 480: “of” -> “by”
Line 485: omit “rather”

Adapted in the text, page 18.

Line 495-498: In Table 2 it appears that the cell cycle time is the same for all cell types in the simulation. Has cell-type dependent cell cycle times been examined in this model?

Cell cycle times were not measured in embryonic livers; this requires dual or cumulative labeling of the cells by injection nucleotide analogs in the pregnant females. Instead, we measured the percentage of proliferating cells. This can be measured either by immunodetection on sections of cell cycle markers (as was performed in our manuscript), or by detection of expression of cell cycle markers in single-cell transcriptomic analyses. The latter approach was followed by Yang et al. (Hepatology 2017; this paper is cited) who found fractions of proliferating cells similar to those that were measured in our experiments.

We did not a priori specify cell-type dependent cycle times in our simulations. We first choose a cell cycle time of 24 hours for hepatoblasts (see Table 2). In a second step we choose the balance between cell cycle progression and quiescence for the cholangiocytes following the description in the last paragraph of the section "Configuration of the in-silico embryonic liver system", page 20, such that the experimentally observed fractions of proliferating hepatoblasts and proliferating cholangiocytes are reproduced. This may result in differences in the apparent cell cycle time. Hence in principle we could conclude a cell cycle time for cholangiocytes by measuring it in our simulations but this parameter is not needed for the conclusions of the paper.

Line 502: omit “net”

This has been adapted in the text, page 18.

Page 19

Line 514: I wonder about the description of this as a pressure. It may be a constraint on expansion derived from a bounding membrane in the embryo, in which case the “pressure” depends on the amount of material within the bounded region and the pressure is not constant. Or it may be simply resistive forces (including momentum) that require force to overcome if the region is to expand, but in the absence of expansion there is no force or “pressure” (described as “mechanical resistance” in the manuscript line 514. Ultimately the model appears to show that this “pressure” either must be balanced by osmotic pressure in the forming duct, or this pressure could be zero and be paired with zero osmotic pressure in the forming duct.

Thank you for this comment: The boundary conditions imposed by the neighboring cells may be a combination of different effects including for example mechanical normal and shear stress, which needs to be overcome by the bile duct lumen pressure. We summarized these in a normal stress (pressure) alone. This background pressure may not be constant over time but large fluctuations seem unlikely as these would destabilize the forming hollow structure, which is why we assume it to be constant and base our simulations on the estimate of intra-tissue pressure measurement in the liver of 50 Pa. A constant pressure over the time course of one day also simplifies our hypothesis testing as it avoids overlay of multiple effects that would have to be dis-tangled aposteriori.
If future measurements would indicate a more complex behavior of such background forces - as significant fluctuations - we could readily adapt the model to such new information.

Line 553: “they” is ambiguous? Do you mean cholangiocyte to hepatoblasts?

The right term is “hepatoblast”. This has been corrected in the text, page 20.

Page 20
Line 554: “delimits” -> “contacts”
Line 559: omit “the same”
Line 559-561: sentence is poorly worded and confusing.

This has been corrected in the text, page 20.

Line 562 vicinity: Perhaps include a table listing the models (0 -> 3) with columns for the model components that are included or omitted in each model?

We included this in the text, page 20.

Line 564: “ into” -> “in”
Line 565-568: poorly written

This has been adapted in the text, page 20.

Line 573: “indicate now” à “show”
Line 574: (Fig 7D) -> (Fig 7C and 7D)?

This has been corrected in the text, page 20.

Line 577: pressure preventing a further growth -> pressure preventing further growth
Line 579: omit the first “apical”
Line 584: omit extra “after”

This has been corrected, page 20

Line 602: “would” -> “could”

Corrected in the text, page 22.

Page 22
Fig 8A (also applies to the other similar graphs): The caption should describe what the plots mean. Solid line is the average response (or a simulation that is closest to the average response) and the shaded areas are the high and low responses from 5 replicates, or the 95th percentile or …?

These are min-max values. This has been clarified in the text, for fig 7,8,9.

Line 629: “guiding towards” -> ”that suggest”

This has been corrected in the text, page 24.

Page 23
Fig 9A: the ordering of the series is odd; 12h, 1h, 2h. The colors don’t have enough contrast.

Figure 9 has been modified, page 24.

-->

Line 642: omit “approach”

Line 643:-645 “This consists in a single-cell agent-based approach, in which every cell is represented in high detail, permitting to take into account the physical forces that determine the cell shape and motion.” à “This consists in a single-cell agent-based approach, in which every cell is represented in spatial detail, taking into account the physical forces that determine the cell’s shape and motion.”

Line 645-647: “The forces that play an essential role in lumen formation embryonic development allegedly are those who originate from (1) pure cell division, (2) apical constriction, or (3)”

à “The forces that play an essential role in lumen formation during embryonic development allegedly are those that originate from (1) pure cell division, (2) apical constriction, or (3)”

Page 34:

Line 648: “The goal was to quantify to which level they can influence the lumen formation.”

à “The goal was to quantify the extant that each influences lumen formation.”

Line 649: “build” à “built”

Line 653-654: “occurred by” à “was implemented as”

Line 654: tissue cells à tissue, cells

Line 655: continuously à continuous

Line 658: distinguished à included

Line 659: “in a” à “to a”

Line 660: “acquiring the capacity to signal to neighboring hepatoblasts. Hereby, our model” à “acquiring the capacity to signal neighboring hepatoblasts. Our model”

Line 663: “rule out” à “implement” (you later establish they contribute but are not sufficient)

Line 664: “sampling of simulation runs over a period of 24 hours”

à “sampling of simulation runs each modeling 24 simulated hours”

Line 667: warrant à give

Line 672: omit “eventually”

Line 673-674: “As in ref. [64], we hypothetise here that the cholangiocytes adapt the excretion of ions to mechanical cues.” à “As in ref. [64], we hypothesize that the cholangiocytes excrete ions in response to mechanical cues.”

Line 674-677: “(iv) The cell-to-cell signalling period controlling the time needed of a cholangiocyte to induce differentiation of an adherent hepatoblast may play are role in the rate of lumen formation, but does not affect its final size.” à “(iv) The cell-to-cell signalling period controls the time needed for a cholangiocyte to induce differentiation of

an adherent hepatoblasts, this may play a role in the rate of lumen formation, but does not affect the lumen's final size."
Line 679: One could, for example, add (add commas)
Line 688: omit "case" to make the caption consistent with the others.

<-------------------

The discussion has been thoroughly revised upon request of the reviewers. When relevant with this new version all comments from reviewer 1 (line 642 to 688) have been addressed. Please see the new discussion with highlighted corrections.

Page 25:
Line 697: I am having trouble viewing Video 6. Using VLC Medi Player (version 3.0.12) the image breaks up for several seconds at about the 2 second and 16 second marks.

We have tested this issue and found that on some combination of platforms and players there are indeed some problems, however not on all. We have created a new mp4 file and kindly ask the reviewer if the problem persists in this video. If so, the longer AVI version can be found on following link:

https://drive.google.com/file/d/1GcCgdQ97L3yt46qgRmypBzHZAhpsfy3b/view?usp=sharing

Supplement S1

The coding language and/or platform is not described.
It is unfortunate that the set of codes and models are not being shared. There is not possible to verify that the biological and mathematical descriptions in the manuscript and supplement are properly instantiated in the code. For that reason, I have not closely examined the mathematical formalisms in the supplement.
A brief discussion of simulation run times would be informative. In the full 2.5D model how long does a single replicate run take on what type of hardware (GPU, multi-CPU, multi-thread, …)

We added technical information as requested. The simulations have been performed with the software TiSiM that will be described in a future communication in more detail. We understand that the reviewer would like to have access to the original sources but the code integrates a number of other running and not yet published long-running projects that cannot be separated out. Students and postdocs working on these projects

could lose their work in case the sources would be opened. We think that markup languages will permit a future interface to run models on codes without opening these. In an earlier test run inside a small consortium we noticed that our personnel is far below the threshold that would it make possible to offer service for our code to the community.

We have further added information of the running time of the simulation, see the supplementary material, page 9

Page 3:
"In the simulations, cells grow by increasing their volume and surface and they can divide when their actual volume reaches the double of the initial value." Isn't the cell division event triggered by a target volume or a normal volume? In the simulation the cells initially start at normal volumes. So "cells divide when their volume reaches twice their normal size" not twice their initial size.

We have adapted the text in SI, page 3 and hope this eliminates the confusion.

"The orientation of cell division can be chosen randomly" I would expect cells (in the absence of a cell-direction such as basal-apical axis) to divide on a plane that is perpendicular to the cell's major axis instead of along an arbitrary plane.

The text on SI page 3 is a generalization, meaning that the direction in which a cell divides, is to be chosen within the model. The user needs to specify whether random or directional cell division is enforced.

Page 4:
"After a certain relaxation time a new global force equilibrium is reached between the nodes." I do not believe there is a way to establish this as a "global force equilibrium". What method is used to avoid being trapped in local minima?

The reviewer is right, the equilibrium is a priori not a global one. We have omitted this in the text.

Page 7 bottom:
Do you observe any discontinuities at the CBM DCM boundaries? The Maugis-Dugdale formulation, and more importantly the exact implementation of that approach, is not guaranteed to be artifact-free at the boundaries between the two cell model types.

We do not observe discontinuities at the interface of CBM and DCM. No separation of DCM and CBM cells will occur unless a force is applied to separate them. There are minimal discretisation and finite mesh size effects (of the DCM), inline with what is usually observed in discretizations. Small differences do not influence the dynamics as the interface is not a “critical” zone in the simulations. The key importance is that proper momentum conservation is established between the CBM and DCM, which is automatically fulfilled by construction of the Maugis Dugdale implementation.

Reviewer #2: My expertise is not in biomechanics and I therefore focus my comments on agent-based model implementation and analysis. The authors present an agent-based model of bile duct lumen formation. The model builds on prior work and systematically explores biological hypotheses. The work is well-presented and detailed. However, some clarifications would help the reader evaluate their findings and put them into context.

• *One key feature of ABMs is stochastic behavior. Most results (e.g. in the idealized system) all seem to show representative images but it is not clear how consistent these behaviors are? I.e. what is the impact of stochastic behavior over multiple simulations with the same parameter set?*

This is an important item which we are regularly asked for. Multicellular systems are self-averaging, meaning that in general, the more cells are considered the less the influence of stochasticity there is on the observables (e.g. see the discussion in Jagiella et. al., Plos Comput. Biol. 2016, Fig. S11 (SI)).

The animation pictures that are shown in for example Fig 8 B-C, are indeed assumed to be representative simulation snapshots for a certain parameter set. Different simulations with an initial different random seed but with the same parameter sets will induce different pictures. However, we did not put these pictures in the document. However, the influence of the random seed is reflected in the lumen area curves (e.g. Fig 8 A). The curves show the maximum and minimum of the lumen.

Some variation is shown in figs 7 and 8 for the full model but it is not stated what the shaded regions represent.

In Figs. 7 and 8, the shaded regions are the minimal and maximal values of the 5 curves generated with the same parameter set but with different seeds. The confidence intervals or Standard deviation could be smaller than given by the bands.

The authors give good motivation and citations for parameter estimates in Table 2. However, there is no uncertainty analysis to quantify how parameter uncertainty would impact their conclusions. This would be another contributor to variation in their simulation results.

We thank the reviewer for this important comment. We first note that there are parameters which were a priori fixed by the geometry of the system, for example the portal vein radius or the background pressure of the tissue. However, these cannot be seen as absolute values for this system as e.g. the portal vein radius found may vary

and the background pressure has to our knowledge not been measured. For the cell-specific parameters, we did not perform a whole parameter sensitivity analysis of each parameter in Table 2:

1. Some parameters in Table 2 were fixed by the experimental observation (e.g. division time, cell radius).

2. Some of the parameters in Table 2 for example, all cell friction coefficients and the cell motility, were chosen based on nominal or physiological values known from literature see for example in the works,

   Drasdo D, Hoehme S, Block M. On the Role of Physics in the Growth and Pattern Formation of Multi-Cellular Systems: What can we Learn from Individual-Cell Based Models. Journal of Statistical Physics. 2007;128:287–345,

   Van Liedekerke P, Neitsch J, Johann T, Alessandri K, Nassoy P, Drasdo D. Quantitative agent-based modeling reveals mechanical stress response of growing tumor spheroids is predictable over various growth conditions and cell lines. PLoS Computational Biology. 2019;doi:10.1371/journal.pcbi.1006273,

   Van Liedekerke P, Neitsch J, Johann T, Warmt E, Gonzàlez-Valverde I, Hoehme S, et al. A quantitative high-resolution computational mechanics cell model for growing and regenerating tissues. Biomechanics and Modeling in Mechanobiology. 2020;doi:10.1007/s10237-019-01204-7.

   These are not specific for the experiment. However, from test simulations in various systems we know that a variation in their physiological range in this system will not affect the mechanical force balance much.

There are also cell specific mechanical parameters which may influence the force balance in the studied bile-duct system, and thus may potentially modify the lumen dynamics. The most obvious ones may be the cell cohesion energy and the cell stiffness.

We have performed new simulations here to illustrate the effect of those parameters.

First, we have performed a simulation set in which we assume a higher global adhesion energy between the cells, but whereby the cadherin density on the apical poles of the cells remains negligibly low. We find that an overall higher adhesion energy between the cells modifies the forces needed to separate cells and thus the origin of the lumen and evolution of its size. The effect of a two times higher global adhesion on the lumen size is shown below in (Fig 10A). As could be expected, a higher adhesion energy decreases the lumen size compared to the nominal adhesion E value. This is a consequence of the higher forces between the cells that need to be overcome by the osmotic pressure.

We now also verified the case where local adhesion would be higher than expected, i.e. at the apical sides of the cells. In particular, we studied the question whether the observed low density of cadherins on the apical side compared to the cell to cell junctions are key to a lumen initiation. To this end, we now ran simulations in which the apical specific adhesion energy was increased from W_{ap} = 10^{-6}J/m^2 to the global nominal adhesion energy (W_{ap} = $9e-4 J/{m}^2$) between the cells. This mimics the scenario in which one would observe a homogeneous, isotropic cadherin distribution on the cell surface. The result shown in (Fig. 10B-C) now shows an overall significant drop of the lumen size. However, the results are lumen pressure dependent. If a low or nominal value of the lumen pressure were assumed, no lumen developed. If a higher lumen pressure was assumed P_l=70 Pa, a lumen developed in several realizations indicating that at sufficiently large lumen pressures, a lumen again forms (Fig. 10C). This suggests a critical importance of the balance between apical adhesion energy and osmotic pressure: if the adhesion forces between the apical sides of the cells would be too high, lumen initiation may be impeded, hence preventing normal bile duct development.

(See page 26.)

Secondly, we performed simulations in which the cells have different mechanical properties. We looked here at the case in which the cells are stiffer. For this we set double values of the cortical stiffness and the volume control stiffness, compared to the nominal values. The results do not show a significant effect on lumen size, which is why we do not show them.

The absence of an effect on the lumen size can be explained by that cortical and volume control stiffness only affect internal forces inside the cell, but not the ones between the cells, the latter being most relevant for the lumen size control.

Generally, although the precise values for each of the parameter are not known, taking values compatible with physiological ranges displays directions of whether combinations of parameter values are compatible with a robust lumen formation which

we found is the case. Moreover, changing parameters in a certain direction permits qualitative prediction of the consequences of such a change even if the precise parameter value may not be known.

• For mechanism I in the idealized system – the difference between the two cell layers are the that one proliferates and the other doesn't at all. When in the data it showed that both layers proliferated but at different rates…does this difference in assumption impact their findings or is it just dependent upon relative proliferation between the two cell layers?

Indeed, mechanism I will likely only impact on the bile duct lumen initiation if the cell proliferation as in the Figure 5 is very disbalanced (e.g. entire proliferation on one side, no proliferation on the other). The disbalance must be significant and not interfere with the cells in the neighborhood of the forming bile duct in order to enable lumen formation.

• Hydrostatic pressure is generated on cell surface triangles based on their contact with tracer particles. Does this pressure depend on the time in contact with tracer particles? E.g. could a triangle become 'marked' if a tracer particle diffuses over its surface and therefore is only in contact for a short period of time? Or asked a different way…how long does the osmotic pressure last? Do these 'marks' from the tracer particles accumulate to account for ion concentrations?

One short contact of the tracer particle is sufficient to mark a free triangle region: the tracer particle is a manner to identify the borders of the lumen similar to having marked test particles in the fluid. The time to mark the entire lumen is short compared to the simulation time or the time scale for a cell division. In our model a triangle becomes unmarked if that triangle regains a contact with another cell's triangle. The marks do not accumulate for ion concentrations in this model, although such a mechanism could in principle be implemented.

Once a lumen has been established it is robust in the simulation as long as the lumen borders do not become permeable.

• The discussion and conclusions largely re-state the results sections and fail to connect their results to broader literature. E.g. are there any known genetic factors associated with bile duct dysfunction that is involved in any of the processes they describe? Do any of their 'failed' lumen formation studies resemble in vivo observations in malformation or disease? What studies would be helpful in the near

future to take advantage of their observations? Are there any other developmental processes that are known to operate in similar ways?

We modified the discussion and included a paragraph on human diseases (Alagille syndrome and HNF1B deficiency) that are characterized by aberrant bile duct formation. We also discuss the mouse models that were generated to investigate these diseases and underline how our modeling impacts our understanding of the disease mechanisms:

---------------->

From line 693 on: "To verify those ..."

To verify the effects of individual physical forces originating from cell division, apical constriction or osmosis, we have first built an isolated, minimal system of adhering cells freely floating in a liquid medium. Our simulations in such an idealized system have shown that each of the three mechanisms can indeed induce lumen formation, provided that the cells are polarized and each of the mechanisms is properly oriented, which in the model was implemented as a polarization vector providing a direction. However, in a real embryonic tissue, cells feel continuous "background forces" from the other growing cells. To take these forces into account an in-silico model mimicking the tissue micro-architecture around the portal vein has been built, in which the various cell types, namely mesenchyme, cholangiocytes and hepatoblasts, have been included. The underlying assumption was that the bile duct originates from a single hepatoblast that differentiates into a cholangiocyte, thereby acquiring the capacity to signal to neighboring hepatoblasts. Our model parameters were informed by the experimentally observed proliferation rates of the hepatoblasts and cholangiocytes. Similar to the minimal system, three submodels have been proposed that implement the individual effects of the three mechanisms. From the sampling of 24h simulation runs the conclusions were: (i) directed cell division alone can initiate a cavity but cannot maintain it during 24h; (ii) including apical constriction in the cholangiocytes improves initial cavity formation but did not give a stable lumen growth. In both cases the background pressure forces are too high and induce a collapse of the cavity; (iii) directed cell division combined with apical constriction and induced osmotic effects of the cholangiocytes creates a stable lumen provided that the osmotic pressure has approximately the same magnitude as the background pressure of tissue. A too low osmotic pressure resulted in a collapse of the cavity whereas a too high one resulted in an unrealistically large growth of the lumen. We hypothesize here that the cholangiocytes excrete ions that serve as signaling molecules; (iv) the cell-to-cell signaling period (the time between sending the signaling and responding to it) controls the time needed for a cholangiocyte to induce differentiation of an adherent hepatoblast. This may play a role in the rate of lumen formation, but does not affect the lumen's final size. In a former ordinary differential equation-based model, Gin and co-workers found a control of cyst lumen size in vitro when elastic tensile stress balanced osmotic pressure whereby cell proliferation occurs in response to lumen expansion Gin et al 2010. This mechanism is largely in line with our findings. Lumen formation in our work is studied within the framework of a single-cell model which permits to include cell-cell adhesion forces and cell-to-cell signalling.

Cell signaling time, which impacts temporal control of differentiation of hepatoblasts to cholangiocytes, also emerged from the modeling as a key regulator of lumen formation. This is not surprising since differentiation consists in acquisition of cell-type specific form and function, and these include the above-mentioned polarization and secretory capacity of the cholangiocytes. The overarching role of differentiation highlighted in our modeling is supported by the analyses of congenital malformations of the bile ducts in humans. Congenital ductopenia, or paucity of the bile ducts, is indeed observed in rare human diseases, Alagille syndrome being the best studied among them. It is characterized by the absence of bile duct formation and results from aberrant Notch signaling consecutive to mutations affecting the *JAGGED1* or *NOTCH2* gene \cite{Mitchell2018}. Mouse models knockout for Notch signalling effectors enabled to identify a critical lumenogenic role of hepatoblast to cholangiocyte differentiation \cite{Zong2009}. Such mouse models also underline the importance of Notch-mediated control of cholangiocyte polarity in shaping the architecture of the epithelium lining the ducts and determining lumen size and maintenance \cite{Andersson2018}. Since Notch signaling stimulates expression of genes normally located at the apical pole of the cholangiocytes, including the chloride transporter CFTR, it is likely that deficient lumen formation associated with perturbed Notch signaling results in part from perturbed osmosis. Moreover, Notch signaling functions also by stimulating expression of the transcription factor *HNF1b*, a transcription factor known to control polarity genes \cite{Zong2009,Poncy2015}, thereby establishing a molecular cascade between Notch, differentiation and polarity. Mice knockout for *Hnf1b* display aberrantly-shaped cholangiocytes and enlarged and irregular lumen at the onset of bile duct formation, but these lumina eventually collapse leading to absence of well-defined bile ducts, as in patients with deficient *HNF1b* gene \cite{Coffinier2002,Roelandt2012}. Whether this relates to abnormal osmosis was not determined, but again is very likely. Together, these observations support the importance of our modeling to test hypotheses explaining how bile ducts may fail to form in human disease.

Our DCM is built in a modular fashion such that cellular detail can be added or removed easily. One could, for example, add components representing an internal cytoskeleton, or add more degrees of freedom to the structure, to obtain a more accurate representation of cell shape. Although these will inevitably bring more complexity to the model, they may become necessary when studying problems such as bile canaliculi formation which demand a finer scale.

- How do the simulated time courses (e.g. of lumen area) compare to in vivo results? Specifically, is an increase in lumen area from zero to ~350um^2 in 24 hours (fig. 9A) realistic?

To address this question we measured 10 lumen areas near the hilum of the liver at E16.5 and at E18.5. At E16.5, the mean area was 14.7 um², ranging from 7 to 36. At E18.5, the mean area was 274.4 um², ranging from 116 to 674 um². Therefore, a value of ~350 um² in 24 hours fits reasonably well within the observed range. This information is provided in the revised caption of Fig. 9A.

• For Figure 4 and Hypothesis III: the figures show results for E16 and E18 but only E16 is discussed in the text. The authors just state that the figures show that these transporters are being expressed at E16 but there is no baseline or control experiment with which to compare. E.g. is there a time point prior to bile duct formation where you would expect these transporters to not be active? Without some reference or control these results are difficult to interpret.

The six genes tested (Cftr, Slc4a2/Ae2, Aqp1, Aqp8, Abcb1/MDR1, and Slc4a4/Nbc1) were selected because of their essential role in bile secretion. Indeed, as summarized by Tabibian *et al.* (Compr. Physiol 2013; this paper is cited), bile secretion by cholangiocytes starts with excretion of Cl^- by CFTR into the lumen. This then activates the Cl^-−/$HCO3^-$ exchanger SLCA42/AE2, which excretes HCO_3^- into the lumen in exchange of Cl^-; this is osmotically followed by efflux of water into the lumen, which requires aquaporins, namely AQP1 and AQP8. This is why we considered the expression of CFTR, SLC4A2/AE2, AQP1 and AQP8 to be necessary to generate osmotic pressure. All four proteins are located at the apical membrane of cholangiocytes. In addition, ABCB1/MDR1 is a broad specificity transporter, and Slc4a4/Nbc1 transports one Na^+ and three HCO_3^- into the bile duct lumen; both are located at the apical membrane and their secretory activity is expected to contribute to osmotic pressure within the lumen.

The expression of the 6 genes in cholangiocytes purified at E16.5 data should be compared with their expression at E18.5. Indeed, published data indicated that bile flows already at E17.5 (Tanimizu et al. Hepatology 2016; 64,175-188), meaning that E18.5 is a time point where bile secretion is active and which may serve as a reference point. Our data in Fig. 4 indicate that the 6 genes tested were expressed in the same range at E16.5 and E18.5 in purified cholangiocytes, meaning that the expression at E16.5 is relevant for bile secretion. We state this more clearly in the revised version.

• Grammatical errors throughout need to be corrected.

We apologize for the errors and we thoroughly revised the text.

Reviewer #3: This is an impressive interdisciplinary paper focussing on modelling aspects of bile duct networks. Specifically investigating the biophysical mechanisms at work during initial bile duct lumen formation during embryogenesis.
The authors use two coupled individual-based, force-based models - a Center-Based Model (CBM) and a Deformable Cell Model (DCM) in order to do this. This approach allows the modelling of both cell-cell interactions and lumen formation in biliary morphogenesis.
The DCM in this paper further develops and extends their previous sophisticated modelling approach through the inclusion of three novel features: (i) Apical-Basal Polarity in each cell; (ii) the modelling of Tight Junctions (TJ) between cells; (iii) Tracer Particles (TP) mimicking osmotic effects.
The authors investigate three different mechanisms hypothesised to contribute to bile duct lumen formation, looking at the individual effects of each of the proposed mechanisms, namely: (i) coordinated cell division; (ii) apical constriction, and (iii) osmotic effects. The computational simulations show that each of these mechanisms can create a lumen in an idealised system without boundary conditions.
Next, guided by the quantification of morphological features and expression of genes in developing bile ducts, the authors construct an in silico system representing a part of the lobule containing the portal vein and surrounding tissue. Using this architecture, the authors use their individual-based model to simulate the effects of the above three mechanisms, both individually and also combined. The results of the computational simulations show that it is necessary for these mechanisms to be coupled together in order to create an initial lumen and then further lumen growth.
The results of the computational simulations have shed light on the underlying biological system (bile duct lumen formation) in a way that would have been very difficult to determine via experiments alone.
The paper is an excellent example of quantitative, predictive and insightful modelling and I recommend publication.

We thank the reviewer for his/her laudatory comments.

There are some minor typographical errors which should be corrected:

Abstract: This model permit realistic simulations --> This model permits realistic simulations
pg. 4, line 83 hypothetised --> hypothesised
pg. 5, line 97: Université catholique de Louvain --> Université Catholique de Louvain

We apologize for these errors and have corrected the manuscript.
