# Supplementary material for: Quantitative modeling identifies critical cell mechanics driving bile duct lumen formation
Source: PLoS Comput Biol. 2022 Feb 18;18(2):e1009653. doi: 10.1371/journal.pcbi.1009653 (PMC8856558; doi:10.1371/journal.pcbi.1009653)
Supplement: Attachment — Submitted filename: PCOMPBIOL-D-21-00553_review_final.docx [file pcbi.1009653.s009.docx]

Quantitative modeling identifies critical cell mechanics driving bile duct lumen Formation
PCOMPBIOL-D-21-00553

April 13, 2021

Page numbers used below are for the manuscript (shown at bottom of page as e.g., 12/30), not the page number in the PDF.

Overall, the work is well thought out and implemented, and addresses interesting issues in lumen formation in bile ducts in developing liver. The manuscript is well structured though clearly not written by a native English speaker. The language is often difficult to follow, or the word choice is poor.

# Main Manuscript

Throughout the document the quote marks are incorrect. If this was typeset in LaTex then the proper quoting characters are on the left `` (two apostrophes) and on the right ‘’ (two single quotes). There are other quoting methods available in latex to give the proper “smart quotes” that point in the proper direction.

A brief discussion in the “Computationl Models” section describing these models as being “2.5D” would be helpful. It is clear in the supplement (and paramter table, and suggested by the figures) that the model is 3D but consist of a single layer of cells constrained to not migrate from the XY plane, but that should be made clearer in the main manuscript.

Page 4

Near lines 57-60. Should cite <http://www.ncbi.nlm.nih.gov/pmc/articles/pmc5221598/> as an example of a spatially defined model where a cavity forms, in this paper it is a cyst but the early process appears relevant to bile duct formation.

Line 65: “aforementioned conditions” is unclear, multiple things have been described in the previous paragraph. Which specific conditions?

Line 83:

In this work, we have put centrally three different mechanisms that are hypothetised to contribute to lumen formation.

🡪In this work, we have included three different mechanisms that are hypothesized to contribute to lumen formation.

Line 87-:

In a second stage, guided by the 87

quantification of morphological features and expression of genes in developing bile ducts 88

specifically in embryonic mouse liver we constructed an in silico system representing a 89

part of the lobule containing the portal vein and surrounding tissue (Fig 1B, D).

🡪 In a second stage, guided by the 87

quantification of morphological features and expression of genes in developing bile ducts 88

specifically in embryonic mouse liver, we constructed an in silico system representing a 89

portion of the lobule containing the portal vein and surrounding tissue (Fig 1B, D).

Page 6

Table 1: the second row of the table appears to list two different antibodies or perhaps the maker is Merck-Millipore?

Page 7

Line 154-155: sentence is poorly written.

Lines 161-166: besides division on a random axis, or related to a polarization axis, what about division perpendicular to the long axis of the cell? A growing cell will often expand in the direction of least resistance giving an elongated cell that generally divides perpendicular to the long axis.

Lines 167-168: very poorly worded and unclear. “compartments” means “cells? “mostly be readily be estimated” should be “mostly be readily estimated”?

Lines 174-175: the polarity vector may not align with a reasonable cell division plane (see previous comment).

Page 8

Fig 2 caption: “mother envelope. upon confinement of the daughter cells,” is there missing text here? This can be written better.

Page 9

Line 185 omit “bonds”

Line 193 shrinked 🡪 shrank or reduced

Line 200-202: sentence it unclear

Line 209: extra space “Fig 2D) . The”

Line 209: the “belt” region is part of the cell’s lateral surface.

Line 210: physical 🡪 discrete

Line 211: “can be secreted by a cell Fig 2E .” 🡪 can be secreted by a cell (Fig 2E).

Line 214: pressures 🡪 pressure

Line 215-217: unclear, perhaps “Conceptually similar, TP could also represent signaling molecules that are sensed by a nearby cells.”

Line 215-217: I don’t think you mention at what stage of development flow starts in both the portal vein and in the bile ducts. Is it possible the TP is bile or a similar material?

Page 10

Table 2: “personal communication” generally includes the person’s name, which may be one of the authors, or someone unrelated to the work in question. Perhaps replace with “estimated”?

Line 240: Use of a period in “lhs.” and “rhs.”. Here there are periods but earlier without, see lines 149 and 151 where it is “rhs”.

Page 11

Line 250: extra )

Line 263: “Beta-catenin is here used as epithelial marker.” 🡪 “Beta-catenin is here used as an epithelial marker.”

Page 12

Line 294: difference may be responsible fot a lumen formation. 🡪 difference may be responsible for lumen formation.

Line 300-302: poorly written sentence. Perhaps “The heterogeneous morphology and circumferential expression of

E-cadherin in hepatoblasts did not allow us to delineate the apical and basal sides or measure the length of the poles.”

Page 13

Line 306: “side of the cell” 🡪 “side of the cholangiocyte cells”

Line 307: as mentioned earlier, possible early secretion of bile micelles instead of salts? Fluorescein is an excellent marker of bile and brightly labels bile canaliculi in mature rodent livers. (<https://pubmed.ncbi.nlm.nih.gov/25339682/> ) Have mice livers of this stage of development been examined for bile formation?

Line 321: Is it possible that flow has begun from bile canaliculi precursors?

Figure 4: bars are StDev? Range?

Page 14

Line 348 Inconsistent use of a period in “Fig. 1A”

Line 355: “Four cells in the upper layer are selected, able to proliferate.” 🡪 “Four cells in the upper layer are selected as being able to proliferate.”

Line 371: “chosen to be random corresponding to a uniform distribution” 🡪 “chosen from a random uniform distribution”

Page 16

Line 388 “with apical constriction, also Tight Junctions (TJ) are present.” 🡪 “with apical constriction, Tight Junctions (TJ) are also present.”

Line 396 “forming” 🡪 “formed”

Page 17

Lines 429-433: perhaps “Here the presence of tight junctions

between the cells helps prevent bursting as they reinforce the cell-cell adhesion

and provide a larger mechanical resistance to osmotic forces. Moreover, the tight

junctions prevent ions from moving through the cell-cell boundary regions thereby hindering leakage

out of the formed cavity.”

Line 438: omit the first “different”.

Line 439: “hypothesised mechanisms in an idealized system with no external constraints to a” 🡪 “hypothesised mechanisms in an idealized system, with no external constraints, to a”.

Line 440: “it is studied” 🡪 “we examine”

Line 443: “aligning” 🡪 “lining”

Line 444: “cues such” 🡪 “cues, such”

Line 445: “signalling has” 🡪 “signalling, has”

Line 457: omit “cut”

Line 459: “warranted”🡪 “obtained”

Line 461: omit “later forming the portal triads”

Line 462 “to” 🡪 “of”

Line 472: “aligning” 🡪 “lining” (this occurs in a couple places)

Line 473-474: is there blood flow in the portal vein at E16.5 – E18.5?

Page 18

Line 480: “of” 🡪 “by”

Line 485: omit “rather”

Line 495-498: In Table 2 it appears that the cell cycle time is the same for all cell types in the simulation. Has cell-type dependent cell cycle times been examined in this model?

Line 502: omit “net”

Page 19

Line 514: I wonder about the description of this as a pressure. It may be a constraint on expansion derived from a bounding membrane in the embryo, in which case the “pressure” depends on the amount of material within the bounded region and the pressure is not constant. Or it may be simply resistive forces (including momentum) that require force to overcome if the region is to expand, but in the absence of expansion there is no force or “pressure” (described as “mechanical resistance” in the manuscript line 514. Ultimately the model appears to show that this “pressure” either must be balanced by osmotic pressure in the forming duct, or this pressure could be zero and be paired with zero osmotic pressure in the forming duct.

Line 553: “they” is ambiguous? Do you mean cholangiocyte to hepatoblasts?

Page 20

Line 554: “delimits” 🡪 “contacts”

Line 559: omit “the same”

Line 559-561: sentence is poorly worded and confusing.

Line 562 vicinity: Perhaps include a table listing the models (0🡪3) with columns for the model components that are included or omitted in each model?

Line 564: “ into” 🡪 “in”

Line 565-568: poorly written

Line 573: “indicate now” 🡪 “show”

Line 574: (Fig 7D) 🡪 (Fig 7C and 7D)?

Line 577: pressure preventing a further growth 🡪 pressure preventing further growth

Line 579: omit the first “apical”

Line 584: omit extra “after”

Line 602: “would” 🡪 “could”

Page 22

Fig 8A (also applies to the other similar graphs): The caption should describe what the plots mean. Solid line is the average response (or a simulation that is closest to the average response) and the shaded areas are the high and low responses from 5 replicates, or the 95th percentile or …?

Line 629: “guiding towards” 🡪 ”that suggest”

Page 23

Fig 9A: the ordering of the series is odd; 12h, 1h, 2h. The colors don’t have enough contrast.

Line 642: omit “approach”

Line 643:-645 “This consists in a single-cell agent-based approach, in which every cell is represented in high detail, permitting to take into account the physical forces that determine the cell shape and motion.”
🡪 “This consists in a single-cell agent-based approach, in which every cell is represented in spatial detail, taking into account the physical forces that determine the cell’s shape and motion.”

Line 645-647: “The forces that play an essential role in lumen formation embryonic development allegedly are those who originate from (1) pure cell division, (2) apical constriction, or (3)”

🡪 “The forces that play an essential role in lumen formation during embryonic development allegedly are those that originate from (1) pure cell division, (2) apical constriction, or (3)”

Page 34:

Line 648: “The goal was to quantify to which level they can influence the lumen formation.”
🡪 “The goal was to quantify the extant that each influences lumen formation.”

Line 649: “build” 🡪 “built”

Line 653-654: “occurred by” 🡪 “was implemented as”

Line 654: tissue cells 🡪 tissue, cells

Line 655: continuously 🡪 continuous

Line 658: distinguished 🡪 included

Line 659: “in a” 🡪 “to a”

Line 660: “acquiring the capacity to signal to neighboring hepatoblasts. Hereby, our model”
🡪 “acquiring the capacity to signal neighboring hepatoblasts. Our model”

Line 663: “rule out” 🡪 “implement” (you later establish they contribute but are not sufficient)

Line 664: “sampling of simulation runs over a period of 24 hours”
🡪 “sampling of simulation runs each modeling 24 simulated hours”

Line 667: warrant 🡪 give

Line 672: omit “eventually”

Line 673-674: “As in ref. [64], we hypothetise here that the cholangiocytes adapt the excretion of ions to mechanical cues.” 🡪 “As in ref. [64], we hypothesize that the cholangiocytes excrete ions in response to mechanical cues.”

Line 674-677: “(iv) The cell-to-cell signalling period controlling the time needed of a cholangiocyte to induce differentiation of an adherent hepatoblast may play are role in the rate of lumen formation, but does not affect its final size.”
🡪 “(iv) The cell-to-cell signalling period controls the time needed for a cholangiocyte to induce differentiation of an adherent hepatoblasts, this may play a role in the rate of lumen formation, but does not affect the lumen’s final size.”

Line 679: One could, for example, add (add commas)

Line 688: omit “case” to make the caption consistent with the others.

Page 25:

Line 697: I am having trouble viewing Video 6. Using VLC Medi Player (version 3.0.12) the image breaks up for several seconds at about the 2 second and 16 second marks.

# Supplement S1

The coding language and/or platform is not described.

It is unfortunate that the set of codes and models are not being shared. There is not possible to verify that the biological and mathematical descriptions in the manuscript and supplement are properly instantiated in the code. For that reason, I have not closely examined the mathematical formalisms in the supplement.

A brief discussion of simulation run times would be informative. In the full 2.5D model how long does a single replicate run take on what type of hardware (GPU, multi-CPU, multi-thread, …)

Page 3:

“In the simulations, cells grow by increasing their volume and surface and they can divide when their actual volume reaches the double of the initial value.” Isn’t the cell division event triggered by a target volume or a normal volume? In the simulation the cells initially start at normal volumes. So “cells divide when their volume reaches twice their normal size” not twice their initial size.

“The orientation of cell division can be chosen randomly” I would expect cells (in the absence of a cell-direction such as basal-apical axis) to divide on a plane that is perpendicular to the cell’s major axis instead of along an arbitrary plane.

Page 4:

“After a certain relaxation time a new global force equilibrium is reached between the nodes.” I do not believe there is a way to establish this as a “global force equilibrium”. What method is used to avoid being trapped in local minima?

Page 7 bottom:

Do you observe any discontinuities at the CBM DCM boundaries? The Maugis-Dugdale formulation, and more importantly the exact implementation of that approach, is not guaranteed to be artifact-free at the boundaries between the two cell model types.
